# Recent Advances in Luminophores for Enhanced Electrochemiluminescence Analysis

**DOI:** 10.3390/molecules29204857

**Published:** 2024-10-13

**Authors:** Zhihan Han, Hao Ding, Dechen Jiang

**Affiliations:** State Key Laboratory of Analytical Chemistry for Life Science, School of Chemistry and Chemical Engineering, Nanjing University, Nanjing 210023, China; hanzhihan1011@126.com

**Keywords:** electrochemiluminescence detection, high-luminescent luminophores, intramolecular luminescent luminophores, spectral-dependent luminophores, biosensing

## Abstract

Electrochemiluminescence (ECL) detection is widely applied in many fields, including chemical measurement, biological analysis, and clinic tests, due to its high sensitivity. Currently, the fast development of many new electrochemical luminophores is continuously improving the ECL-based detection ability. Besides the enhancement of luminescence emission for a high detection sensitivity, minimizing the effect of co-reactants on ECL detection and achieving multiple analysis in one sample are also the main directions in this field. This review focuses on a summary of recently prepared new luminophores to achieve the three aims mentioned above. Especially, the review is composed by three parts, focusing on the luminophores or materials with high ECL efficiency, self-enhancing properties, and multi-color ECL luminophores. The fabrication of biosensors using these molecules is also reviewed to exhibit the advances in biological applications.

## 1. Introduction

Electrochemiluminescence (ECL) is a light emission process that includes the redox reactions on the electrode surface under a certain voltage. Since the luminescent reagent is excited and emits light without the need for any other light source, ECL has the advantages of a high sensitivity and low background signal [1,2,3]. Accordingly, since its discovery, electrochemiluminescence (ECL) technology has received great attention from the scientific community and has been widely applied in the fields of biological analysis, environmental monitoring, food safety, and medical diagnosis [4,5,6,7,8,9]. Typically, the annihilation and co-reaction pathways are two popular mechanisms for ECL, in which the co-reactive pathway is mostly used [10,11,12,13]. In this pathway, the luminescent reagent (e.g., ruthenium complex) is oxidized or reduced at the electrode, and the co-reactants (e.g., tri-n-propylamine or hydrogen peroxide) help the luminescent reagent to generate excited-state substances and provide additional electrons or reaction intermediates [14,15]. The intermediate is energetically unstable, and thus, it returns to the ground state, releasing energy and emitting visible light in the form of photons. The involvement of co-reactants not only improves the luminescence efficiency but also increases the sensitivity. It is noted that the presence of co-reactants can still lead to some negative effects, such as increased background noise and instability of the ECL signal.

Luminophores and co-reactants, electrode materials, pH, potentials, etc., have a great impact on ECL generation and the performance of ECL analysis [16,17,18,19], among which luminophores are the most important component in ECL detection. These luminophores with different luminescence efficiencies determine the sensitivity and effective detection range of ECL analysis. Therefore, obtaining new luminophores with high ECL efficiency is always the first task in this field. Thanks to the rapid development of materials science, ECL detection technology is experiencing an unprecedented development momentum, with more emerging high-luminescent ECL luminophores [20,21,22]. Due to the continuous enhancement of ECL emission, this method is now being applied for the analysis of living bio-samples, such as single living cells [23,24,25,26]. However, in these studies, toxic co-reactants become a problem, so their concentrations should be minimized. To address this challenge, self-enhancing ECL emission has emerged as a new direction that will push the application of ECL-based analysis into biological study. Furthermore, to achieve detection in complex samples, simultaneous multiplex ECL analysis in a single sample is another important direction, which relies on the preparation of new luminophores to give the spectrum-resolved signals. According to these new directions in ECL-based analysis, this review summarizes the recent development on the ECL luminophores, focusing on high luminescence, the intramolecular ECL process, and multicolor ECL emission. Related applications are also reviewed to exhibit their significant contributions to biological studies.

## 2. New Luminophores with High ECL Efficiency

In either co-reaction or an annihilation ECL system, the luminescent material with high ECL efficiency plays a core role in the development of ultra-sensitive ECL sensors. The ECL quantum efficiency of classic luminophores such as tris(2,2′-bipyridine)ruthenium(II) (Ru(bpy)_3_^2+^) and luminol is relatively low, which cannot meet the needs of ultrasensitive ECL analysis. With the development of nanotechnology and quantum technology, many luminophores have been used to achieve enhanced ECL emission. This section focuses on the recent development of luminophores toward achieving high ECL efficiencies.

### 2.1. Semiconductor Nanocrystal Quantum Dot

**Figure 1:**
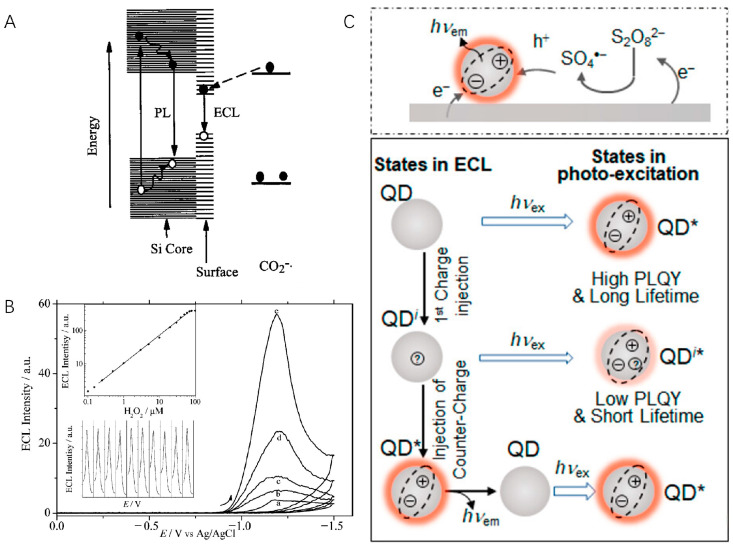
(**A**) ECL and PL mechanisms of Si nanocrystals. Copyright 2002 American Association for the Advancement of Science [29]. (**B**) ECL detection of H_2_O_2_ by using CdSe QDs-modified electrode. The ECL intensity increased with increasing H_2_O_2_ concentration from 0 μM to 10 μM (a–e). Inset: calibration curve (top) and reproducibility (down). Copyright 2004 American Chemical Society [34]. (**C**) Illustration of QD cathodic ECL generation (**top**) and states (neutral QD (QD), singly charged intermediate (QD^i^), and excite state (QD*)) involved in ECL and PL (**bottom**), in which symbols on QDs represent different charged states. *hν*_ex_: excitation light. *hν*_em_: emission light. Copyright 2020 American Chemical Society [40].

Quantum dots (QDs) are a special type of nanomaterial that have a size between 1 and 10 nm and unique optical and electronic properties [27,28]. Traditional QDs usually refer to nanocrystals (NCs) made of semiconductor materials (such as CdSe, PbS, CdTe, etc.), which are used as promising luminescent materials. In 2002, Bard et al. reported the first case of ECL generated from silicon NCs (diameter 2 to 4 nm) [29]. Both annihilation and co-reactant ECL from NCs were observed, which exhibited a peak maximum at 640 nm. This work demonstrated the chemical robustness of Si NCs upon hole and electron injection and the possibility of using NCs as ECL luminophores. Note that the ECL spectrum has a significant red shift of 200 nm from the band-edge photoluminescence (PL), which was later proved to be surface-state emissions (Figure 1A) [30,31]. These surface defects formed by surface atoms result in the quench of band-edge luminescence process and low quantum yield. By passivating the surface states of NCs, the same group observed ECL emissions from both surface states on the NCs and from the bulk in NCs [32]. After this revolutionary work, QDs quickly became an ideal material to achieve high-efficiency ECL since they are efficient emitters with high quantum yields and size-tunable color. 

In 2004, Weller et al. reported the first bandgap ECL of CdSe/CdS NCs in aqueous solution, which opens the avenues of QDs applications into practical biosensing [33]. By depositing CdSe NCs on a paraffin-impregnated graphite electrode (PIGE), Ju et al. achieved the first QD ECL sensor for the detection of H_2_O_2_ [34]. Based on the electron transfer reaction between electrochemically reduced NC species and hydrogen peroxide, the fabricated ECL sensor could detect the co-reactant H_2_O_2_ with a detection limit of 100 nM (Figure 1B). Apart from H_2_O_2_, a lot of inorganic and organic molecules and ions, such as Cu^2+^, dopamine, and glucose (with the help of glucose oxidases), can be detected by directly quenching or enhancing the ECL signals of QDs [35,36,37]. Zhu et al. modified CdS NCs, gold nanoparticles, and a low-density lipoprotein (LDL) ligand on the electrode and generated strong ECL in solution [38]. The LDL concentration can be measured in a label-free manner through the decrease in ECL intensity upon the specific binding of LDL to its ligand. In addition, using QDs as ECL tags in classic ECL assays, proteins, and nucleic acids can also be quantified [39].

Although QDs have been applied in ECL analysis for years, most aqueous QDs generate ECL from surface state, which is indicated by red-shifted wavelengths, broad emission spectrum, and low ECL quantum efficiencies relative to PL [27]. To address this problem, Su, Qin, and Peng et al. prepared CdSe/CdS/ZnS core/shell/shell quantum dots (Figure 1C) [40]. By engineering the interior inorganic structure and the inorganic–organic surfaces, the obtained QD exhibited an absolute ECL efficiency of 99%, which is quite close to its near-unity photoluminescence QY. The ECL intensity was reported to be six orders of magnitude higher than that of Ru(bpy)_3_^2+^ under the same conditions [41]. Recently, they further modulated the surface ligand of CdSe/CdS/ZnS QDs and observed an increase of ECL intensity by 100 times when changing the ligand from oleate to acetate [42]. These results are expected to provide guidance on the design of QDs for analysis applications.

### 2.2. Metal Nanoclusters

Metal nanoclusters (NCs) have become attractive ECL emitters due to their unique compositions with atomic precision, rich luminescence characteristics, and well-defined electrochemical features [43]. Ding and coworkers reported the ECL of a series of gold clusters, including Au_25_, Au_38_, and Au_144_ [44,45,46]. These gold nanoclusters display a near-IR emission, ranging from 800 to 930 nm, that is similar to their PL, indicating that they originate from the same or related excitation states. In addition, smaller NCs (Au_25_ and Au_38_) show stronger ECL than the larger one (Au_144_). A high relative ECL efficiency of >350% in reference to that of Ru(bpy)_3_^2+^/TPrA was obtained with Au_38_. By rational structure design, Au_21_(SR)_15_ (where SR is H, CH_3_, C_2_H_5_, and C_2_H_7_) and bimetallic Au_12_Ag_13_ nanoclusters even revealed a relative ECL efficiency of 270 and 400%, respectively [47,48]. In both cases, the improved properties can be attributed to the structure–function correlations. For example, the 13th Ag atom at the central position is the key in Au12Ag13 nanoclusters, which stabilizes charges on the LUMO orbital and makes the cluster core rigid.

Although excellent ECL generation was observed, it should be noted that these works were all performed in organic solutions. For better ECL bioanalysis applications, such as cell and tissue imaging, Wang et al. reported the first intense ECL from aqueous soluble AuNCs nanoclusters [49]. By covalently attaching co-reactant N,N-diethylethylenediamine (DEDA) onto Au clusters, oxidative reduction ECL arises from intracluster reactions with a maximum relative ECL efficiency of 17 compared with that of Ru(bpy)_3_^2+^. Effective strategies such as pre-oxidation and aggregation-induced emission have been reported to enhance the ECL intensity of Au clusters [50,51,52]. For instance, the ligand-induced assembly of CuNCs simultaneously facilitates electrochemical excitation and radiative transition of NCs, thereby improving their ECL efficiency [52]. However, the intrinsic ECL intensity of nanoclusters in aqueous solution is weak, and therefore, the ECL efficiency of these aqueous soluble nanoclusters cannot outperform Ru(bpy)_3_^2+^.

### 2.3. Carbon-Based Nanomaterials

**Figure 2:**
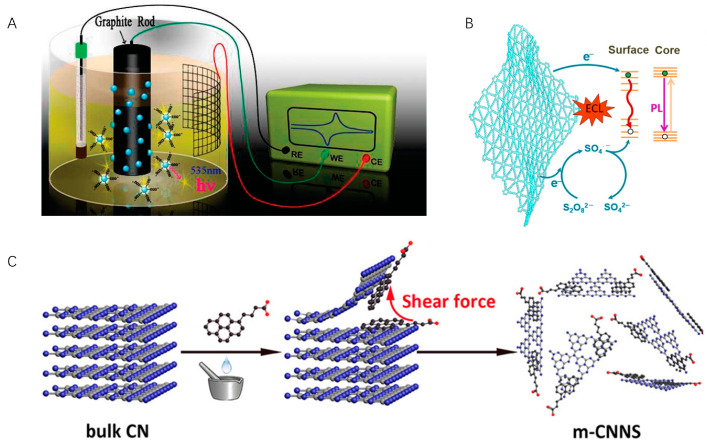
(**A**) Schematic illustration of the synthesis of water-soluble CNCs. Copyright 2009 American Chemical Society [55]. (**B**) ECL mechanism of GDY. Copyright 2022 WILEY-VCH [59]. (**C**) Exfoliation and modification of 2D carbon nitride. Copyright 2017 American Chemical Society [61].

Carbon-based nanomaterials have the advantages of high abundance, low toxicity, and good biocompatibility, making them a highly sought-after ECL luminescent material. Carbon dots, graphene QDs, and graphitic QDs have also been explored [53,54]. Chi et al. studied the ECL behavior and mechanisms of water-soluble carbon nanocrystals (CNCs) for the first time (Figure 2A) [55]. Using S_2_O_8_^2−^ as the co-reactant, the ECL response of CNCs was observed. In this case, CNCs were prepared via electrochemical oxidation of a graphite working electrode and had abundant -COOH groups at the surfaces, which benefits their labeling. This top-down method possesses advantages including easy operation and mass production but suffers from low product yield.

In contrast, the bottom-up method prepares carbon nanomaterials from molecular precursors, which grow up to form “quantum-sized” particles. This strategy enables the precise control of particle morphology and size, and aggregation can be avoided. Common building blocks include ascorbic acid, glucose, fructose, etc. [56,57,58]. Recently, using hexaethynylbenzene as the precursor, Zhang and Mao et al. fabricated graphdiyne (GDY), a new 2D carbon allotrope for ECL generation (Figure 2B) [59]. Different from other carbon allotropes, GDY comprises both sp^2^- and sp-hybridized carbon and is an intrinsic semiconductor material. In the presence of K_2_S_2_O_8_, GDY produces strong ECL emission at 705 nm in aqueous media, and the ECL efficiency reaches 424% compared with Ru(bpy)_3_Cl_2_/K_2_S_2_O_8_.

Graphitic carbon nitride (CN) is another two-dimensional carbon nanomaterial with adjustable molecular structure and electronic properties [60]. Previously, the poor dispersibility of CN in solvents (similar to nanotubes and graphene), low efficiency of CNs exfoliation, and original chemical inertness limit their practical applications. Therefore, tremendous efforts have been devoted to developing CN-based ECL luminophores. Zhang et al. proposed a non-covalent modification and exfoliation methodology to prepare two-dimensional CNs (Figure 2C) [61]. They mechanically grind bulk CN with aromatic molecules, obtaining CN nanosheets (m-CNNS) noncovalently modified with 1-pyrenebutyrate. DNA and other biological recognition moieties can thus be covalently conjugated onto functionalized CNs via EDC/NHS activation. Moreover, using bottom-up methods such as thermal condensation, a series of CN films were prepared on the electrode surface [62]. A transparent polymeric film on an FTO electrode exhibits more than 2000 times higher ECL intensity than that of the reference Ru(bpy)_3_Cl_2_/K_2_S_2_O_8_ system, which enables the preparation of an ultrasensitive visual DNA biosensor using the naked eye [63].

### 2.4. Organic Nanoparticles

Organic nanoparticles are also an important type of nanomaterials with ECL signals. Tunable optical properties, easy functionalization, and excellent biocompatibility are the main advantages of organic nanoparticles as ECL luminophores. In 2008, the ECL imaging analysis of single conjugated polymer nanoparticles was achieved after a long integrating time (10–40 s) [64]. Different kinds of polymers have been used to prepare nanoparticles, while their ECL efficiency is lower than that of general metal complexes [65,66]. The poly[2-methoxy-5-(2-ethylhexyloxy)-1,4-(1-cyanovinylene-1,4-phenylene)] Pdots gave a relative ECL efficiency of ca. 12% (vs. Ru(bpy)_3_^2+^/TPrA) [67,68]. Ju et al. used Pdots as emitters to modify electrodes [68]. Compared with the Ru(bpy)_3_^2+^-modified electrode, the relative ECL efficiency was calculated to be 120%. Using this Pdot as an ECL tag in immunoassay, the limit of detection for cytokeratin-19-fragment detection was reduced to 0.12 pg mL^−1^. To date, the research on organic NPs is just beginning. Challenges such as poor water solubility and relatively low ECL efficiency need to be addressed.

## 3. Self-Enhancing ECL Luminophores

To obtain a high ECL signal, most ECL systems require a co-reactant. However, classic co-reactants such as TPrA are toxic, corrosive, and volatile but need to be used in high concentrations. Some co-reactants produce byproducts during the electrochemical reaction process, increasing the background noise and reducing the detection sensitivity. Meanwhile, the selection of co-reactants might change the response of the ECL system to the target, thereby affecting the accuracy of the detection. Many researchers have begun to consider in situ generation of co-reactants to reduce the impact of exogenous co-reactants. Einaga et al. used boron-doped diamond (BDD) electrodes to promote the conversion of inert SO_4_^2−^ into co-reactant S_2_O_8_^2−^ [69]. In another work, a BDD electrode facilitated the oxidation of carbonate (CO_3_^2−^) into peroxydicarbonate (C_2_O_6_^2−^), which further reacted with water to produce co-reactant H_2_O_2_ [70]. Although powerful, the BDD electrode is not a general solution applicable for ECL analysis. To avoid the influence of exogenous co-reactants, more studies should focus on self-enhancing luminophores, whose co-reactant is integrated into the luminophore structures. According to the type of ECL luminescent center (molecules or nanoparticles), we classify these luminophores into two categories.

### 3.1. Molecules as the Luminescent Center

**Figure 3:**
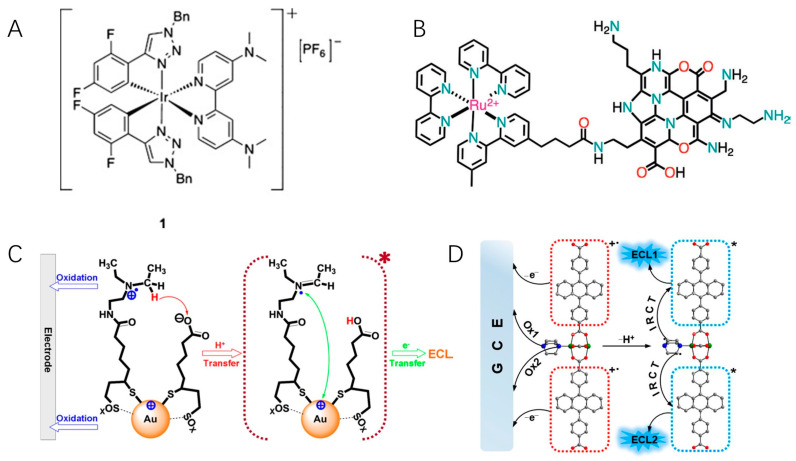
(**A**) The self-enhanced iridium(III) complex with two dimethylamino (dma) groups. Copyright 2012 WILEY-VCH [72]. (**B**) The covalently linked hybrid of NCNDs and Ru(bpy)_3_^2+^. Copyright 2017 WILEY-VCH [77]. (**C**) ECL mechanism of self-enhanced Au clusters. Copyright 2016 American Chemical Society [49]. (**D**) Illustration of the stepwise ECL mechanism of self-enhanced MOFs. Copyright 2021 American Chemical Society [80]. The asterisk * means excited state.

In 1996, Martin et al. observed that ruthenium-labeled penicillin molecules could generate ECL in the absence of any amine, which results from the intramolecular electron transfer from penicillin to ruthenium [71]. Zysman-Colman and Ding reported for the first time the ECL auto-enhancement phenomenon. They synthesized an iridium(III) complex bearing aryltriazole C^N ligands with two dimethylamino (dma) groups (Figure 3A) [72]. Three emissions were observed in spooling spectrum, correlating to the typical annihilation route (543 nm) and dma co-reactant route (608 and 651 nm). Compared with a structurally similar complex without dma substituents, the ECL intensity and efficiency in the absence of co-reactants were self-enhanced by 16 and 5.5 times, respectively. The strategy of integrating co-reactant and luminophore in a single molecule opens a new avenue for simplifying ECL detection protocols and enhancing detection sensitivity. Two ruthenium (II) complexes carried Schiff base cavities as the luminophore [73]. Thanks to the electrochemical oxidation of phenolic hydroxyl groups and the resonance structure of the imino radicals, electrons can be transferred within the molecule, thereby achieving effective ECL without the addition of co-reactant TPrA. After connecting a dimethylamino moiety to cationic diaza [4] helicene by a short linker, the first self-enhancing organic ECL dye was obtained [74]. In combination with a series of organometallic complexes, self-enhanced multi-color ECL was achieved as well.

Amine-rich polymers and nanomaterial co-reactants can be also used as self-enhancing moieties. Zhuo and Yuan et al. combined the poly(ethylenimine) (PEI) and a derivative of Ru(bpy)_3_^2+^ bearing -COOH groups to prepare self-enhanced nanocomposites (PEI-Ru) [75,76]. An immunoassay was ultimately devised by using the nanocomposite as ECL tags, with an enhancement in ECL intensity up to 17 times and a detection limit as low as to 0.3 fg/mL. Nitrogen-doped carbon nanodots (NCNDs) have been demonstrated to be powerful alternatives to TPrA. By covalently linking luminophores and NCNDs, De Cola and co-workers synthesized a hybrid of NCNDs and Ru(bpy)_3_^2+^, in which NCNDs function as both carriers and co-reactants (Figure 3B) [77]. Interestingly, the ECL emission intensity of the Ru-NCND hybrid is not only higher than that obtained for Ru(bpy)_3_^2+^ but also higher than that of the uncoupled Ru(bpy)_3_^2+^/NCND system. The reason might be that intramolecular electron transfer is more efficient than the intermolecular transfer, in which the former favors shorter electron transfer distance and less energy loss. A boron carbon nitride nanosheets–Ru nanocomposite (BCN NSs-Ru) was successfully employed to construct an ECL mRNA sensor [78]. No co-reactants were used, and the limit of detection was 32.3 aM.

### 3.2. Nanomaterials as the Luminescent Center

As described before, amine moieties can be modified onto nanomaterial surfaces or embedded into their structures to obtain self-enhanced ECL. Wang et al. covalently attached the co-reactant N,N-diethylethylenediamine (DEDA) to Au nanoclusters, enabling ECL generation from Au cluster without additional and high-excess co-reactants (Figure 3C) [49]. The intracluster reactions reduce the complication of mass transport between Au cluster and co-reactants and significantly enhance its ECL intensity. By directly embedded tertiary amine groups as co-reactants into the side chain of the polymer unit, a self-enhanced Pdots ECL system (TEA-Pdots) was obtained [79]. The ECL intensity of TEA-Pdots was 132 times higher than that of the mixture of Pdots and TEA at equivalent, and the ECL efficiency waws 4 times higher than that of [Ru(bpy)_3_^2+^]/TEA system. By using this highly efficient nanoparticle as an ECL tag, ECL microimaging of membrane protein in single living cells without additional co-reactants was achieved. Similar to the design of Pdots, self-enhanced metal–organic frameworks (MOFs) can be facilely prepared by integrating co-reactants as building blocks in MOFs (Figure 3D). Different from the above works, luminophores and co-reactants are not covalently linked in self-enhancing MOFs. The luminophore 9,10-di(pcarboxyphenyl)anthracene (DPA) and co-reactant 1,4-diazabicyclo[2.2.2]octane (D-H_2_) were integrated as two ligands in Zn^2+^ MOFs [80]. By shortening the distance between DPA and D-H_2_ by the frameworks, an efficient charge transfer can be realized between the emitter and co-reactant. Studies have shown a 26.5-fold increase in ECL intensity compared with that of the mixture of DPA and D-H_2_.

## 4. Multi-Color ECL Luminophores

Multiplex analysis capable of determining multiple analytes in a single complex sample has become increasingly popular in recent years. The simultaneous detection not only saves detection time and samples but also gives more information about the sample and improves the accuracy of detection. Typically, approaches to achieve parallel ECL detection can be roughly divided into three categories: potential-, spatial-, and spectrum-resolved strategies. Potential-resolved ECL measurements rely on luminophores with potential-controlled switch on/off properties, while only a few luminophores have been reported. Spatial-resolved assays are usually expensive since well-designed spot arrays are required. In comparison, spectrum-resolved ECL can be facilely achieved in one solution with diverse multi-color ECL luminophores, which provides the possibility of multiplex immunoassays. In this part, we mainly focus on the emerging spectrum-resolved ECL systems, some of which may exhibit potential- and spatial-resolved features as well.

### 4.1. Transition Metal Complexes

The ECL of Ru(bpy)_3_^2+^ and its derivatives is generated by the radiative decay of metal-to-ligand charge transfer, and thus, it is difficult to tune their ECL colors for multiplex detection. Over the last few decades, new molecular luminophores have been reported and studied in-depth. The most important one is the iridium(III) complex. Compared with Ru(bpy)_3_^2+^, iridium complexes generally exhibit much higher PLQY. Thanks to an increased ligand field stabilization energy, the ECL wavelengths of iridium complexes can be facilely modulated in the region from visible to near-UV by changing the substituents on their ligands and coordination modes. These advantages facilitate multianalyte detection and make it a promising option in ECL analysis.

**Figure 4:**
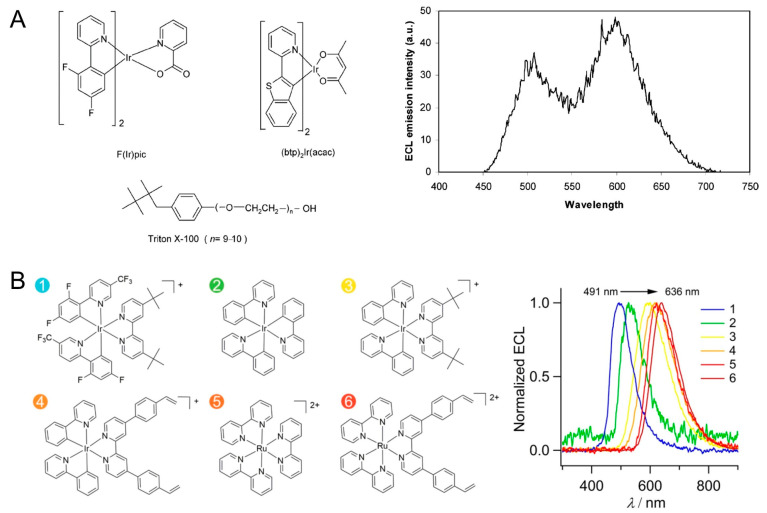
(**A**) Structures of multicolor ECL luminophores and the ECL mission spectrum of F(Ir)pic and Ru(bpy)_3_^2+^ in a same solution. Copyright 2004 American Chemical Society [81]. (**B**) Molecular structures of six ECL luminophores (1–6) and their ECL spectra. Copyright 2018 American Chemical Society [88].

Richter et al. studied the multicolored ECL from a mixture of Ir and Ru complexes (Figure 4A) [81,82]. Ir complexes with blue (Ir(dfppy)_2_(pic), λ_ECL_ = 470 nm), green (Ir(ppy)_3_, λ_ECL_ = 517 nm), and red (Ir(btp)_2_(acac), λ_ECL_ = 600 nm) emissions were synthesized. Either blue or green emitters can be distinguished from Ru(bpy)_3_^2+^ in ECL spectra, providing the possibility of detecting multiple analytes in a single ECL experiment. Since then, various Ir complexes with different ligands have been prepared, offering more options for spectrum-resolved detections [83,84]. In three-dimensional (intensity, wavelength, and potential) ECL spectra and potential-dependent ECL images, different molecules exploit different ECL onset potentials and wavelengths, which promises to enable multianalyte detection [85,86]. Particularly, Francis and Hogan et al. reported the potential-controlled on/off mechanism of Ir(ppy)_3_, whose ECL can be “switched-off” at high potentials. This molecular has been widely applied in ECL multiplex analysis [87].

Su et al. constructed a potential-resolved multicolor ECL system for multiplex immunoassay (Figure 4B) [88]. They synthesized six Ir(III) and Ru(II) complexes, in which the red, green, and cyan luminophores were loaded into polystyrene beads, encoded with three different detection antibodies, and used for the simultaneous detection of three antigens in a sample volume of 300 μL. Xu and coworkers prepared a closed bipolar electrode (BPE) and employed ECL as an optical reporter [89]. The formation of immunocomplex on the BPE modulated the resistance of BPE and the potential in the reporter pole; therefore, the emission color of the Ru(bpy)_3_^2+^/Ir(ppy)_3_ mixture was finely tuned. By using Irpic-Ome, Ir(ppy)_2_(acac), and Ru(bpy)_3_^2+^ as blue, green, and red ECL emitters to detect three miRNA biomarkers, the ECL emission can cover whole visible regions [90]. It should be noted that iridium complexes suffer from poor water solubility; therefore, the use of polymer beads and bipolar configuration is necessary, in which luminophores are separated from the sensing interfaces. Synthesizing water-soluble iridium complexes and developing new sensing strategies remain hot topics [91,92]. Moreover, although differences in the emission maxima of these metal complexes reach 100–150 nm, there are considerable spectral overlaps. These molecules have a broad spectrum with a full width-at-half-maximum up to ca. 60–80 nm, which limits the throughput of color-resolved ECL analysis in the visible region.

### 4.2. Multi-Color Nanomaterials

**Figure 5:**
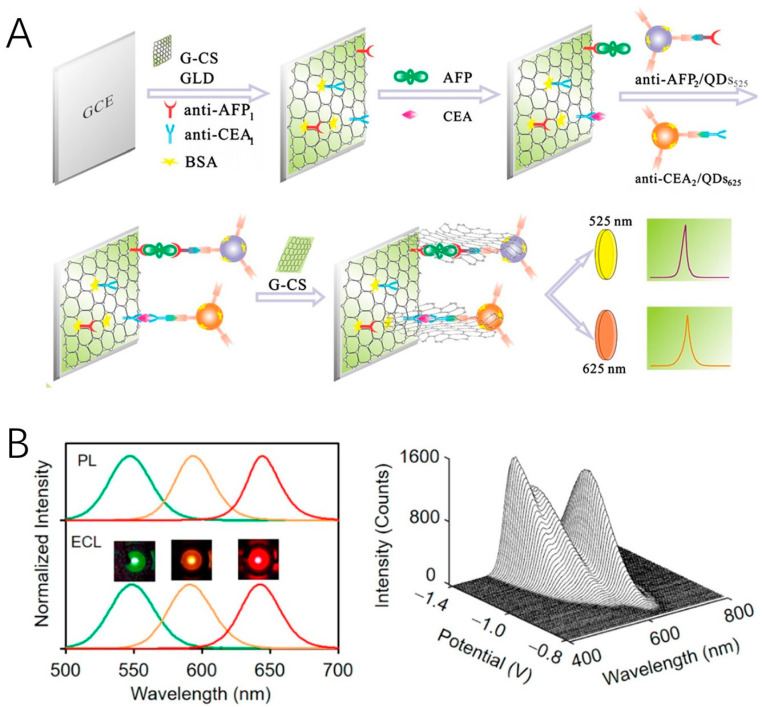
(**A**) Fabrication of the ECL immunosensor using QDs as labels and graphene as conducting bridge. Copyright 2013 Elsevier [95]. (**B**) Multicolor ECL emission from core/shell/shell QDs with different core sizes. Copyright 2020 American Chemical Society [41].

Due to quantum confinement effect, QDs possess size-dependent optical and electronic properties, which inherently favor multi-color ECL analysis [93]. Highly efficient, stable, and spectrally resolved QDs with narrow emission line width are ideal alternatives for developing multiplex ECL assays. CdTe (λ_ECL_ = 776 nm) and CdSe (λ_ECL_ = 550 nm) QDs were employed as spectrum-resolved ECL emitters for the simultaneous detection of carcinoembryonic antigen (CEA) and alpha-fetoprotein (AFP) [94]. By establishing a sandwich-type immunoassay on the glassy carbon electrode, extremely low detection limits of 1 pg/mL and 10 fg/mL for CEA and AFP were achieved. In the immunoassay, some QDs labeled on the secondary antibody might not be electrochemically oxidized or reduced on the electrode surface due to the distance. To promote direct electron transfer, Guo et al. used graphene as a conducting bridge between the electrode and QDs, which led to a 30-fold enhancement in ECL intensity (Figure 5A) [95]. An interesting work reported by Pang and coworkers adopted three QDs with emission wavelengths ranging from 525 nm to 625 nm to visualize biomolecules on single cells [96]. These QDs were decorated with glucose oxidase, perfringolysin O, and lysenin protein to recognize glucose, cholesterol, and sphingomyelin, respectively. Molecular dynamics and inter-molecular interactions can be monitored simultaneously.

Note that there are two ECL mechanisms for QD ECL, including surface-state and band-edge ECL generation. The latter one is favored in multi-color since it corresponds to size-dependent ECL wavelengths. Su and Peng et al. used an additional wide bandgap ZnS shell to isolate surface traps and a CdS intermediate layer to relieve the lattice strain between ZnS and CdSe (Figure 5B) [41]. By rationally modulating the size of the CdSe core, a group of CdSe/CdS/ZnS core/shell/shell QDs were synthesized, which exhibited multicolor band-edge ECL with K_2_S_2_O_8_ as the co-reactant. In addition to QDs, Pdots allow for multiplex analysis as well. By doping Pdots with luminol and diethylamine, respectively, Pdots emitting light at 450 nm and 675 nm were prepared [97]. Two microRNAs were quantified with the true color ECL imaging.

To better compare the performance of the different luminophores discussed above, here, we summarize typical ECL sensors based on these luminophores in Table 1. Note that some of the emerging luminophores are at an early stage of development without analytical applications, and we also list them in the table to illustrate their unique ECL properties.

## 5. Conclusions

In this paper, we summarized the recent advances in ECL luminophores, covering the hot topics including new luminophores with high ECL efficiencies, self-enhancing luminophores, and multi-color ECL systems. Ultrasensitive, co-reactant-free, and high-throughput analyses have been accomplished in these works. However, despite the extensive research into new ECL luminophores, most ECL assays in both fundamental and commercial systems still rely on Ru(bpy)_3_^2+^. Challenges remain in this field. First, the application of nanomaterials in ECL analysis is still less studied due to the complexity of their structures and relatively large sizes compared with molecular luminophores. Highly efficient luminophores with well-defined structures, clear ECL mechanisms, excellent electrochemical and optical properties, high water solubility, and biological compatibility are still desired. Secondly, most self-enhancing luminophores were constructed by embedding amine moieties into their structures. Since the co-reactant cannot be regenerated in the ECL process, the intensity and stability of these luminophores should be of concern. Breakthroughs in the design principles of self-enhancing luminophores or novel co-reactants are expected. Finally, restricted by the full width-at-half-maximum of ECL spectra, color-resolved ECL analysis is limited to the proof-of-concept experiment, which determines a maximum of two or three biomarkers. The multiplexing capacity should be further elevated. With the combination of new principles and methodologies, we believe ECL analysis could achieve wider and deeper applications in the fields of bioanalysis, environmental detection, and medical diagnosis.

## Figures and Tables

**Table 1:** Application of different ECL luminophores in ECL sensing *.

Luminophore	Co-Reactant	ECL Wavelength	Target	Dynamic Range	Limit of Detection	Ref.
CdSe QD	H_2_O_2_	-	H_2_O_2_	2.5 × 10^−7^–6 × 10^−5^ M	1.0 × 10^−7^ M	[34]
CdS QD	K_2_S_2_O_8_	-	LDL	0.025–16 ng mL^−1^	0.006 ng mL^−1^	[38]
Ag_2_Se QD	K_2_S_2_O_8_	695 nm	Dopamine	0.5–19 μM	0.1 μM	[98]
CdSe/ZnS QD	H_2_O_2_ and K_2_S_2_O_8_	-	DNA ATP Ag^+^	100 aM–100 pM 10 fM–10 nM 1 pM–1 μM	35.0 aM 3.71 fM 0.28 pM	[99]
Au_25_ NC	K_2_S_2_O_8_	620 nm	Dopamine	2.5–47.5 μM	-	[100]
Au_25_ NC	Triethyamine	680 nm	Pb^2+^	10^−9^–10^−5^ M	-	[101]
Au_21_ NC	TPrA	847 and 874 nm	-	-	-	[47]
Carbon Nitride	K_2_S_2_O_8_	~450 nm	DNA	10^−13^–10^−6^ M	3.6 × 10^−14^ M	[61]
Graphdiyne	K_2_S_2_O_8_	705 nm	-	-	-	[59]
Carbon Dots	tetrabutyl Ammonium bromide	-	Fe^2+^, Fe^3+^	5 × 10^−6^–8 × 10^−5^ M	7 × 10^−7^ M	[58]
Pdots	TPrA	~550 nm	CEA CY211 NSE	1 pg mL^−1^–500 ng mL^−1^	0.17 pg mL^−1^ 0.12 pg mL^−1^ 0.22 pg mL^−1^	[67]
[(dFphtl)_2_Ir(dmabpy)]^+^	-	543, 608, and 651 nm	-	-	-	[72]
PEI-Ru	-	-	MicroRNA-21	10^−16^–10^−11^ M	0.03 fM	[76]
BCN NSs-Ru	-	687 nm	TK1 mRNA	100 aM–1 nM	32.3 aM	[78]
TEA-Pdots	-	-	HER2	-	-	[79]
[Ru(bpy)_2_(dm-bpy-dc)]^2+^ Ir(ppy)_3_ Ir(df-ppy)_2_(ptp)	TPrA	Red Green Blue	-	-	-	[86]
Ir(dFCF_3_ppy)_2_(dtbbpy)^+^ Ir(ppy)_3_ Ru(bpy)_2_(dvbpy)^2+^	TPrA	491 nm 526 nm 636 nm	CEA AFP *β*-HCG	-	-	[88]
CdSe/CdS/ZnS QDs with different core sizes	K_2_S_2_O_8_	549 nm 592 nm 643 nm	-	-	-	[41]
CdTe CdSe	(NH_4_)_2_S_2_O_8_	776 nm 550 nm	CEA AFP	10 pg mL^−1^–10 ng mL^−1^ 50 fg mL^−1^–100 pg mL^−1^	1 pg mL^−1^ 10 fg mL^−1^	[94]

* CY211: cytokeratin-19-fragment; NSE: neuron-specific enolase; HER2: human epidermal growth factor receptor 2; *β*-HCG: beta-human chorionic gonadotropin.
